# Healthcare Professionals' Knowledge of Neonatal Resuscitation in Ethiopia: Analysis from 2016 National Emergency Obstetric and Newborn Care Survey

**DOI:** 10.1155/2019/8571351

**Published:** 2019-07-16

**Authors:** Mulugeta Woldu Abrha, Tsrity Tadese Asresu, Alemnesh Abraha Araya, Haftom Gebrehiwot Weldearegay

**Affiliations:** ^1^Tigray Health Research Institute, Mekelle, Ethiopia; ^2^Mekelle University, College of Health Sciences, Mekelle, Ethiopia; ^3^Mekelle University, College of Health Sciences, Department of Midwifery, Mekelle, Ethiopia

## Abstract

**Background:**

Birth asphyxia, which accounts for 31.6% of all neonatal deaths, is one of the principal causes of neonatal mortality in Ethiopia. Adequate knowledge of newborn resuscitative procedures plays an important role in early diagnoses and suitable management. However, there are limited data on healthcare professionals' knowledge about neonatal resuscitation. Thus, this study aimed to determine the knowledge of healthcare professionals about neonatal resuscitation and factors affecting it.

**Methods:**

Data from the Ethiopian 2016 national Emergency Obstetric and Newborn Care survey of 3,804 health facilities that provided maternal and newborn health services were analyzed. We have included 3804 healthcare providers, who attended the largest number of deliveries in the last month prior to the survey, and assessed their knowledge of neonatal resuscitation. It was also determined whether certain factors were associated with healthcare providers' knowledge through linear regression method.

**Result:**

The overall knowledge score of the healthcare providers about neonatal resuscitation ranged from 12 to 24 out of 37 items (with mean score of 18.4 (±5.47) and mean score percentage of 49%). The findings showed that providers trained on neonatal resuscitation (*β*=2.65, 95% CI: 0.65, 4.62; p <0.00), facilities that had guideline of neonatal resuscitation (*β*=2.50, 95% CI: 0.60, 3.52; p =0.01), and availability of essential equipment (*β*=0.95, 95% CI: 0.44, 1.45; p =0.02) were significantly associated with sufficient knowledge of neonatal resuscitation in Ethiopia.

**Conclusion:**

Overall knowledge of neonatal resuscitation was insufficient. Trained healthcare providers, having guideline on neonatal resuscitation, and availability of essential equipment were significantly associated with knowledge of neonatal resuscitation. Competency and simulation-based in-service training and refresher training complemented by supportive supervision and mentorship are helpful ways to put up providers capability to perform neonatal resuscitation.

## 1. Introduction

The first 28 days of life period constitute the most vulnerable time for a child's survival. Globally 130 million babies are born every year; about four million die in the first 4 weeks of life. Neonatal asphyxia accounts for 20.9% of neonatal deaths. Among the new delivered babies approximately 10% require some assistance to begin breathing at birth, and about 1% requires extensive resuscitative measures. Most neonatal deaths (99%) arise in low-income and middle-income countries [1, 2].

About one-quarter of all neonatal deaths worldwide are caused by perinatal asphyxia. Twenty-three percent of neonatal deaths in low-income countries and 31.6% in Ethiopia are attributed to birth asphyxia. This finding underlines the fact that perinatal asphyxia is still a burden of the world and also in Ethiopia [3–6].

Ethiopia is one of the countries with the highest neonatal mortality in the world and is responsible for 29 deaths per 1,000 live births, which could be 9 times more than that of developed countries, where the rate is 3 per 1,000 live births [7–11]. The effect of birth asphyxia is not limited only to death but also has a short and long term neurodevelopment sequel, including cognitive and motor disabilities which are almost untreatable. Survivors of asphyxia may also develop hypoxic-ischemic encephalopathy, posttraumatic stress disorders, neurologic disability, low cognitive functions, and neurological sequel [12–14].

Proper knowledge of newborn resuscitation can prevent the consequences of perinatal asphyxia. adequate knowledge of resuscitative procedures in the newborn plays an important role in proper, early diagnoses, suitable management, and reducing the number of complications in newborns with life-threatening conditions [15–17]. However, there is limited information on healthcare professionals' knowledge about neonatal resuscitation. Therefore, this study aimed to understand the knowledge of healthcare professionals about neonatal resuscitation and factors affecting it. Moreover, it will have greater input to program managers and policymakers in design, proper implementation, and evaluation of programs on the reduction of under-five children mortality and improvement of children healthcare.

## 2. Materials and Methods

### 2.1. Data Source

We used data from the 2016 Ethiopian Emergency Obstetric and Newborn Care (EmONC) survey [18]. The EmONC assessment was a national cross-sectional census of 3804 facilities of all public hospitals, health centers, and all private facilities (higher-clinic and above) that provided maternal and newborn health services. The EmONC assessment did not include health posts or medium and small clinics.

The survey used 13 questionnaires comprised of 12 health facility assessment modules and one healthcare system assessment module adapted from the Averting Maternal Death and Disability program. One healthcare provider who attended the largest number of deliveries a month before (or the latest month with delivery if there was no delivery in the last month) among all those who are present at the time of the visit was included. Thus, a total of 3804 healthcare professionals (HCPs) were assessed for their knowledge of neonatal resuscitation (NR) service.

### 2.2. Measurement of Knowledge

Knowledge of providers about neonatal resuscitation was determined using a set of 37 Yes/No questions [Table 4]. Each correct answer was valued at one point, and a wrong answer attracted no point. Questions that were not answered were treated as wrong answers. Eventually, participants were then grouped into two categories based on their total score on the knowledge scale: sufficient knowledge (80% or higher) and insufficient knowledge (less than 80%) [18], after the overall cumulative mean score and the cumulative mean percentage score were calculated.

### 2.3. Data Analysis

Data were analyzed using SPSS version 21™ software. Descriptive statistics were used to summarize facility and provider characteristics. Characteristics of the study population were presented as median and interquartile ranges for continuous data with skewed distribution or with mean and standard deviation for variables with normal distribution. The normality of distribution of quantitative variables was tested by Kolmogorov–Smirnov test. We used linear regression analysis method to assess the association between healthcare providers' knowledge of NR service and explanatory variables. Simple linear regression analyses were conducted and those independent variables with p value of ≤ 0.25 were considered for multiple linear regression with the forward likelihood ratio method. Finally, variables with p<0.05 in the multiple linear regression analysis were considered to declare statistically significant associations between covariates and knowledge of NR service. Residual normality assumption was used to assess the model fit.

## 3. Result

### 3.1. Characteristics of Studied Population and Facility

A total of 3804 healthcare professionals were included to the study. The providers median age was 25.6 (IQR=4.75) and around two-thirds of them (n=2411, 63.4%) were females. Based on qualification of providers, the majority of them, 3193 (83.9%), were midwives and mean working experience was around 4 years. Three thousand fifty-five out of 3804 providers were trained on neonatal resuscitation and also above half, 2307 (60.6%), of them reside in urban. Two-thirds of the providers, 595 (66.6%), had copy of their job description.

Regarding facility characteristics, 3481 (91.5%) of the facilities were health centers/clinics and the majority, 3662 (96.3%), of the facilities were owned by government. Around two-thirds, 2479 (65.2%), of the facilities had guideline on NR and 293 (38.2%) of them had staff rotation policy for newborn care service. Around two-thirds, 2517 (66.2%), of the facilities had no separate newborn corner and the majority, 3612 (95.0), of them did not have separate NICU. Health facilities had averagely five infrastructural components out of ten components and six essential equipment items out of the eight components [Table 1].

### 3.2. Knowledge about Neonatal Resuscitation

This study showed that the total average score of knowledge of neonatal resuscitation was 18.4 (SD=5.47) with a range of scores from 12 to 24 and mean score percentage of 49% [Table 2].

### 3.3. Predictors for the Knowledge of Neonatal Resuscitation

To identify the potential predictors between knowledge and providers/facility characteristics, univariable linear regression was fitted with the following independent variables: age of the provider, current qualification of care provider, work experience, providers trained on NR, residence of care provider, copy of job description, facility type, managing authority, facility having guideline on NR, facility having staff rotation policy for newborn care service, facility having a separate newborn corner, facility with separate neonatal intensive care unit (NICU), infrastructure, and essential equipment of the health facilities. The variables that were associated with knowledge score (p<0.25) were included in the multivariable linear regression model.

After adjusting for all other variables, three variables, providers trained on NR, having guideline on NR, and availability of essential equipment, were identified as predictors of knowledge of neonatal resuscitation. Every unit increase in NR training, i.e., changing from untrained to trained, resulted in 2.65 unit increase in the knowledge score (95% CI: -2.80,-0.86; p <0.00). One unit increase in availability of essential equipment resulted in 0.95 point increase in the knowledge score (95% CI: 0.44, 1.45; p =0.001). Facilities that had guideline of NR were 2.50 times knowledgeable when compared with facilities with no NR guideline (95% CI: 0.60, 3.52; p =0.01) [Table 3].

## 4. Discussion

In this study, the overall knowledge of neonatal resuscitation was insufficient, and trained healthcare providers, having guideline on NR, and availability of essential equipment were significantly associated with knowledge of neonatal resuscitation.

The overall knowledge score of the healthcare providers on neonatal resuscitation ranged from 21 to 27 out of 43 items (with mean score of 21.33 (±5.9) and mean score percentage of 49%). This is in line with studies done in Kenya, which indicates that only 35.4% of the participants managed to score above the minimum knowledge competency level [19]; Ethiopia, which shows that the overall knowledge about neonatal resuscitation in health professionals was poor (42.8%) [20]; and Ghana (38%) [21]. This might be due to lack of exposure to an adequate number of real cardiopulmonary resuscitation cases, simulation-based training, updating training, and certification process before graduation. This result shows that there is insufficient knowledge among health professionals about NR and we are yet faced with high numbers of resuscitation attempts largely poorly executed. These findings reflect ailing health system and support the view that very few medical institutions currently provide optimal training on essentials of newborn care.

Other findings showed that healthcare providers trained on neonatal resuscitation were significantly associated with adequate knowledge of neonatal resuscitation. This result was in line with studies done in Afghanistan [22], Zambia [23], Malawi [24], and Ghana [21]. This might be due to lack of exposure to an adequate number of real cardiopulmonary resuscitation cases, simulation-based training, lack regular updates in training, and certification process before graduation, which might lead to poor knowledge of neonatal resuscitation. Thus, simulation-based routine and frequent NR training need to be organized for better retention of the knowledge. Teaching of the basic neonatal care including the neonatal resuscitation should be stressed during the medical education itself to ensure acceptable neonatal outcome.

The study revealed that facilities possessing neonatal resuscitation guideline had significant association with knowledge of neonatal resuscitation. This is similar to a study done in Kenya [25]. This could be due to presence of NR guidelines and action plans at the resuscitation stations ensuring that standardized NR procedures are performed for all newborns born with birth asphyxia. These guidelines will assist healthcare providers and program managers responsible for implementing maternal and child health programs to develop or adapt national or local guidelines, standards, and training materials on newborn care.

Another issue was the relationship of availability of essential equipment with neonatal resuscitation. This result was also reported by studies done in Zimbabwe [26] and South Africa [27]. This could be due to lack of essential neonatal resuscitative equipment that good NR skills are failing to develop, since the acquired knowledge cannot be put into practice and then the acquired knowledge will be lost after not practicing for 6 month.

## 5. Conclusion

The overall knowledge of neonatal resuscitation was insufficient. Trained healthcare providers, having guideline on NR, and availability of essential equipment were significantly associated with knowledge of neonatal resuscitation. Thus, simulation-based routine and frequent NR training need to be organized for better retention of the knowledge. Teaching students about basic neonatal resuscitation procedures during medical education stay will improve their knowledge while they are assigned to the community. Availing essential equipment and specific neonatal resuscitation guidelines could increase the knowledge of health providers.

## Figures and Tables

**Table 1:** Characteristics of studied population and health facility on NR, 2016 (N=3804).

Variables	Values (N=3804)
*Providers characteristics *	
*Age in completed years, Median [IQR] *	*25.6±4.75*
*Sex of care provider, n (*%)	
Female	2411(63.4)
Male	1393(36.6)
*Current qualification of care provider, n (*%)	
MD/HO	145(3.8)
Midwives	3193(83.9)
Nurse	466(12.3)
*Work experience in years, (Mean ± SD) *	*3.94±9.21*
*Providers trained on NR, n (*%)	
No	749(19.7)
Yes	3055(80.3)
*Residence of care provider, n (*%)	
Urban	1497(39.4)
Rural	2307(60.6)
*Provider has copy of their job description *	
Yes	595(66.6)
No	299(33.4)
*Facility characteristics *	
*Facility type, n (*%)	
Hospital/MCH center	323(8.5)
Health center/Clinics	3481(91.5)
*Operating agency, n (*%)	
Government	3662(96.3)
Private	142(3.7)
*Facility has guideline on NR, n (*%)	
Yes	2479(65.2)
No	1959(51.5)
*Facility has staff rotation for new born care service, n (*%)	
Yes	1287(33.8)
No	2517(66.2)
*Facility has a separate new born corner, n (*%)	
Yes	1038(72.2)
No	2766(27.3)
*Facility has separate NICU*	
Yes	192(5.0)
No	3612(95.0)
*Infrastructure components∗,mean(±SD)*	*5.41±1.56*
*Mean availability of essential equipment∗∗(mean ± SD)*	*6.10±1.70*

*∗*Electricity functionality at newborn corner room, water at newborn corner room, generator, telephone, radio, ventilation, toilet, light source, fan/air conditioning, waiting area.

*∗∗*Mucus extractor, neonatal size Ambu (ventilator) bag, infant face masks (sizes 0 or 1), towels/blanket or cloth for newborn, new born resuscitation table, radiant warmer, fetal stethoscope, Mucus trap for suction/suction apparatus.

**Table 2:** Knowledge regarding neonatal resuscitation in health care professionals, Ethiopia.

Domain	Number of items	Score (Mean ± SD)
Immediate new born care practices	16	9.88±2.67
Diagnosis of birth Asphyxia	4	1.82±1.08
Activities after asphyxiated baby	7	3.44±1.65
Critical signs of a newborn baby that need referral	10	3.23±1.85
Knowledge score (mean ± SD)	37	18.4±5.47

**Table 3:** Linear regression analysis of predictors of knowledge of NR among health care providers.

Characteristics	Univariate Analysis			Multivariate Analysis		
	Β	95%CI	P-value	*β* adj.	95%CI	P-value
*Age in completed years, Mean [SD] *	*0.01*	*(-0.03,0.05)*	*0.62*			*NS*
*Sex of care provider, n (*%)						
Female	0.47	(0.11,0.83)	0.00			NS
Male	1					
*Current qualification of care provider, n (*%)						
MD/HO	0.71	-0.20,1.62	0.13			NS
Midwifes	1.65	(1.17,2.12)	0.00			
Nurse	1					
*Work experience in years, (Mean ± SD) *	*-0.020*	*(-0.04,-0.01)*	*0.03*			*NS*
*Providers trained on NR, n (*%)						
No	1					
Yes	1.11	(0.67,1.55)	0.00	2.65	(0.65,4.62)	0.00
*Residence of care provider, n (*%)						
Urban	1					
Rural	-0.99	(-1.34,-0.63)	0.00			NS
*Provider has copy of job description, N=218 *						
Yes	1					
No	0.36	(-0.41,1.13)	0.00			NS
*Facility type, n (*%)						
Hospital/MCH center	-1.55	(-2.17,-0.93)	0.00			NS
Health center/Clinics	1					
*Operating agency, n (*%)						
Government	-0.21	(-1.13,0.71)	0.00			NS
Private	1					
*Facility has guideline on NR, n (*%)						
Yes	1.25	(0.88,1.61)	0.00	2.50	(0.60-3.52)	0.00
No	1					
*Facility has staff rotation for new born care service, n (*%)						
Yes	1					
No	0.51	(0.14,0.88)	0.00			NS
*Facility has a separate new born corner, n (*%)						
Yes	1.16	(0.77,1.54)	0.00			NS
No	1					
*Facility has separate NICU*						
Yes	1.46	(0.66,2.25)	0.00			NS
No	1					
*Infrastructure components∗,mean(±SD)*	*0.24*	*(-0.04,0.52)*	*0.09*			*NS*
*Mean availability of essential equipment∗∗ (mean ± SD)*	*0.58*	*(0.48,0.68)*	*0.00*	*0.95*	*(0.44,1.45)*	*0.00*

NB. *β* adj. is adjusted *β*; P is P-value, CI is confidence interval.

**Table 4:** Questions about health professionals' knowledge of neonatal resuscitation, 2016.

Questions	
What do you do for newborn immediately following delivery?	Deliver the baby skin
	Dry the baby's body
	Cover the baby with dry towel
	Assess the bobby's breathing
	Clamp cord after 1 minutes
	Provide chlorhexidine gel for cord care
	Ensure baby is kept warm (skin)
	Initiate breastfeeding
	Apply tetracycline eye ointment once (with 60 minutes)
	Give vitamin K (after 60 minutes)
	Weigh the baby (after 90 minutes)
	Give BCG
	Give polio 0
	Cord should remain dry
	Apply chlorhexidine for cord care for 7 days
	Give sponge baths until cord falls off

How would you diagnosis birth asphyxia?	Depressed/no breathing
	Floppiness
	Heart rate below 100 beats per minute
	Cyanosis

What do you do after asphyxiated baby?	Call for help
	Explain to mother condition of baby
	Place the newborn face up
	Wrap or cover baby, except for face and upper portion of chest
	Position baby's head so neck is slightly extended
	Clear secretions if seen
	Start ventilation

What are the signs of critical illness for a newborn baby that need referral?	Lethargic
	Comatose
	Seizure
	Unable to feed
	Weak or absent cry
	Excessive cry
	Cyanosis
	Bulging fontanel
	Persistent jaundice
	Respiratory distress

## Data Availability

The data used to support the findings of this study are available from the corresponding author upon request.

## Abbreviations

EmONC: Emergency Obstetric and Newborn Care
MD: Medical doctor
HO: Health officer
NICU: Neonatal intensive care unit
NR: Neonatal resuscitation.
